# Successful public health measures preventing coronavirus disease 2019 (COVID-19) at a Michigan homeless shelter

**DOI:** 10.1017/ice.2020.439

**Published:** 2020-08-26

**Authors:** Dan Kelly, Holly Murphy, Ravi Vadlamudi, Ruth Kraut, Kate Dalessio, Anurag N. Malani, Meghan Glabach, Juan Luis Marquez

**Affiliations:** 1 Shelter Association of Washtenaw County, Ann Arbor, Michigan; 2 St Joseph Mercy Ann Arbor Hospital, Ann Arbor, Michigan; 3 Packard Health Clinic, Ann Arbor, Michigan; 4 Washtenaw County Health Department, Michigan

*To the Editor*—Coronavirus disease 2019 (COVID-19) has spread rapidly in homeless shelters across the United States.^1,2^ An investigation in 5 cities identified 37% and 21% severe acute respiratory coronavirus virus 2 (SARS-CoV-2) positivity among residents and staff, respectively.^3^ In response, the Centers for Disease Control and Prevention (CDC) urged testing all residents and staff of homeless shelters on April 22.^4^

Delonis Center is the only adult shelter for Washtenaw County (population, 350,000) with 5,000 homeless persons countywide, serving >1,100 people annually. Delonis accommodates 60 people per night as a warming shelter and feeds 200 people twice daily. The serviced population is 52% African American and 45% Caucasian (average age, 45 years; 70% male). Notably, 67% report an underlying disability. More than 70% have a comorbidity, including heart disease, chronic obstructive pulmonary disease, seizure disorder, and renal failure. Mental health conditions are noted among 48% and substance abuse among 33%.

Michigan, and particularly southeastern Michigan, was heavily affected by COVID-19 early in the United States, with 65,533 reported cases as of July 5, 2020.^5^ Washtenaw County reported 1,067 cases by April 28 (281 per 100,000 population) and 1,526 cases by July 5, 2020.^5^ We describe our robust COVID-19 infection prevention strategies at Delonis Center with universal testing results and outcomes.

## Methods

The first case of COVID-19 in Michigan was reported on March 10, 2020—the day the governor declared a state of emergency. We implemented our plan on March 13, including symptom screening (ie, new or worsening cough, dyspnea, subjective or measured fever (≥38°C or 100.4°)) before entry with a risk-based triage, social distancing, and secondary housing at local churches or hotels. Clients triaged “green” with negative screen were cleared to sleep at the shelter in regular conditions. Those screened “yellow,” with ≥1 symptom, were triaged to mattresses 2 m (6 feet) apart with surgical masks and underwent a clinical assessment. Those determined ill, or screened “red,” were transported to the emergency room. People under investigation were quarantined in private rooms.

We secured a secondary site to enable sheltering in place on March 24, and we secured an offsite hotel on March 29. We implemented a temporary pay increase for staff and recruited 30 extra staff. On April 8, we mandated masks (surgical or cloth). We extended a warming shelter indefinitely to maintain shelter-in-place for all in need. On April 28 and 29, universal screening and molecular testing for SARS-CoV-2 were offered to all residents and staff.

## Results

From March 13 until April 30, 15,000 health screenings were conducted. In total, we sheltered 113 persons (40%) over age 51 directly at Delonis and 281 persons overall with 4 offsite locations with a nightly average of 135. On average, 160 individuals (38 at a time, socially distanced) were served a warm meal twice daily. At all sites, clients were encouraged to practice social distancing and to shelter in place. Two positive cases were identified. Both cases were screened “red”: the first on March 17 and the second on March 25. On April 28 and 29, molecular testing was performed for 99 residents and 38 staff with 0 positive. As of July 5, there have been no additional cases.

## Discussion

Our protocol was successful in identifying 2 early symptomatic cases, resulting in zero additional cases once universal testing was implemented.

We attribute the success of our program to key interventions.^6^ Symptom screening before entry, conducted multiple times daily, identified the only 2 COVID-19 cases at our facility before widespread transmission could occur. Maintaining the warming shelter and expanding our capacity to shelter all “in-need” early minimized the flow of clients through public places. Onsite medical and psychiatric assessment identified high-risk individuals to prioritize for isolation. We optimized communication within our site with phone meetings 3 times daily and had daily communication with the local public health team.

Our study has several limitations. The success of our implementation was challenged by innate health risks faced by the population served, including mental health conditions and substance abuse. The sensitivity of our screening protocol was decreased by clients presenting intoxicated. In response, we added clinical cues to screen this population. We observed that intoxicated clients were less adherent to social distancing and more likely to have another comorbid medical condition. Alcohol-based hand sanitizer use was inhibited by risk of ingestion by clients and we were limited on sinks. Increased family obligations and self-quarantining strained staff, which we addressed by addition of temporary staff. All of the efforts described required significant unbudgeted expenses. As the COVID-19 pandemic continues, we anticipate difficulty sustaining this level of protection due to funding limitations, team fatigue, and the client flow into the community.

When universal testing was offered, <10% of residents refused COVID-19 testing. Still, no further cases were identified in the following 2 months.

In conclusion, where prior reports of COVID-19 among homeless shelters and other congregate settings have been concerning, our experience is hopeful. Interrupting the spread of COVID-19 in congregate settings poses a great challenge, more pressing as states lift aspects of quarantine. Our outcomes demonstrate that an early and comprehensive COVID-19 preparedness plan may effectively protect a vulnerable homeless population. The reality of homelessness in the United States has become more visible in the COVID-19 pandemic as we assess our capacity to protect the most vulnerable. Any long-term plan should include a commitment to housing for all. In the short term, continued support to extend implementation of COVID-19 infection prevention and control activities, like those we describe here, is imperative. Key aspects of our model may be adapted to other settings to protect vulnerable populations.
